# DICER1 regulated let-7 expression levels in p53-induced cancer repression requires cyclin D1

**DOI:** 10.1111/jcmm.12522

**Published:** 2015-02-20

**Authors:** Xin Sun, Shou-Ching Tang, Chongwen Xu, Chenguang Wang, Sida Qin, Ning Du, Jian Liu, Yiwen Zhang, Xiang Li, Gang Luo, Jie Zhou, Fei Xu, Hong Ren

**Affiliations:** a Oncology Department of the First Affiliated Hospital of Xi'an Jiaotong UniversityXi'an, Shaanxi Province, China; b Georgia Regents University Cancer CenterAugusta, GA, USA; c Tianjin Medical University Cancer Institute and HospitalTianjin, China; d Institute of Radiation Medicine, The Chinese Academy of Medical SciencesNankai District, Tianjin, China; e Department of Cancer Biology, Kimmel Cancer Center, Thomas Jefferson UniversityPhiladelphia, PA, USA; f Department of Breast Oncology, Affiliated Cancer Hospital of Guangzhou Medical UniversityGuangzhou, Guangdong Province, China; g Department of Radioation Oncology, Fudan University, Shanghai Cancer CenterShanghai, China

**Keywords:** let-7, regulatory loop, cyclin D1, DICER1, cell apoptosis, cancer stem cells

## Abstract

Let-7 miRNAs act as tumour suppressors by directly binding to the 3′UTRs of downstream gene products. The regulatory role of let-7 in downstream gene expression has gained much interest in the cancer research community, as it controls multiple biological functions and determines cell fates. For example, one target of the let-7 family is cyclin D1, which promotes G0/S cell cycle progression and oncogenesis, was correlated with endoribonuclease DICER1, another target of let-7. Down-regulated let-7 has been identified in many types of tumours, suggesting a feedback loop may exist between let-7 and cyclin D1. A potential player in the proposed feedback relationship is Dicer, a central regulator of miRNA expression through sequence-specific silencing. We first identified that DICER1 is the key downstream gene for cyclin D1-induced let-7 expression. In addition, we found that let-7 miRNAs expression decreased because of the p53-induced cell death response, with deregulated cyclin D1. Our results also showed that cyclin D1 is required for Nutlin-3 and TAX-induced let-7 expression in cancer repression and the cell death response. For the first time, we provide evidence that let-7 and cyclin D1 form a feedback loop in regulating therapy response of cancer cells and cancer stem cells, and importantly, that alteration of let-7 expression, mainly caused by cyclin D1, is a sensitive indicator for better chemotherapies response.

## Introduction

The let-7 family of miRNAs was traditionally regarded as tumour suppressors, targeting and degrading downstream oncogenes through association with Argonaute to form the RNA-induced silencing complex (RISC) 1,2. Regulatory loops between miRNAs and their targeted genes revealing elaborate networks in cellular regulation are an emerging area of exiting cancer research 3. Decreased let-7 may be the indicators of p53 induced cell apoptosis and inhibition of self-renewal ability of stem cell 1. It has been shown that p53 interacted with cyclin D1 in on many levels 4,5, and that let-7 represses cyclin D1 expression 6,7; however, whether a feedback loop exists between let-7 and cyclin D1, and what mechanisms are involved in this regulation need to be explored. Let-7 miRNAs are generated and processed by the endoribonuclease Dicer 1, through which the pre-miRNAs are processed to generate the 20–22 nucleotide mature miRNAs 8,9. The RNAas III endoribonuclease DICER1 could cleave stem–loop–stem structured pre-miRNA to form the mature let-7 miRNAs, and also was targeted and degraded by mature let-7 miRNAs [Correction added on 21 May 2015 after first online publication: the final statement of this sentence was added]. Previously, two groups noticed that cells lacking cyclin D1 produced less of the miRNA-processing proteins, and therefore had reduced levels of mature miRNA 8,10. However, what relationship exists between let-7 and cyclin D1, and what mechanisms are involved in this regulation remain unknown.

Let-7 could sensitize cancer cells to chemotherapy-induced apoptosis and inhibit the self-renewal ability of cancer stem cells 11,12. Determining how let-7 interacts with cyclin D1 in breast cancer will definitely help us to understand the mechanisms of miRNAs regulated cell biology. Furthermore, the regulatory relationship between miRNAs and their targeted genes challenges the traditional theory that miRNAs regulate mRNAs and then the cell biology uniaxially, suggesting the comprehensive network of gene regulations in cell fate determination. The application of miRNAs detection in clinical diagnosis should be designed and chose more accurately.

## Materials and methods

### Cell culture and the infection of let-7 lentiviral vectors

Human breast cancer cell lines of MCF-7 and MDA-MB-231 were purchased from ATCC and kept at the Central Laboratory affiliated to the Medical College of Xi'an Jiaotong University, China. These cancer cells were kept and cultured in RPMI-1640 medium (Gibco, Thermo Scientific, China), and supplemented with 10% fetal bovine serum (FBS, Thermo Scientific, China), 1% penicillin and streptomycin (Gibco, Thermo Scientific, China). The mammosphers were cultured in DMEM/Ham's F-12 medium (Cellgro, CORNING, China) and supplemented with 10 μg/ml EGF, 10 ng/ml human basic fibroblast growth factor (hbFGF, Lifescience, Roche, China), 10 ng/ml of hydrocortisone, 4 μg/ml insulin, and 1% penicillin and streptomycin (Gibco, Thermo Scientific, China) [Correction added on 21 May 2015 after first online publication: the location of DMEM/Ham's has been inserted and the location of penicillin and streptomycin has been changed.] 13. All cells were cultured in 5% CO_2_ at 37°C. Let-7 miRNAs and CTL-LSC1 (control) encoded in LV-10 (pGLVU6/RFP) and shRNA-cyclin D1 was synthesized and purchased from GenePharma Co.,Ltd (Shanghai, China). The propagation of lentiviral vectors was conducted following published protocols 14,15. Lentiviral plasmids and packaging vectors (PMD2/PSPAX2) were transfected into HEK 293T cells by the Ca^2+^ phosphate transfection method. The viral supernatants were collected 72 hrs after transfection. Cells were infected at approximately 70% confluence in culture medium supplemented with 8 μg/ml polybrene, and later selected with 4 μg/ml of puromycin [Correction added on 21 May 2015 after first online publication: 8 ?g/ml polybrene has been added.]. The cDNA encoding human cyclin D1 was amplified by PCR and the PCR product was blunted and ligated into Nco I site of pENTR4 vector (Addgene ID: 17423). A LR reaction was performed using LR ClonaseTM II enzyme (Life Technologies, USA) by following manufacturer?s manual, which allows the transfer of the cDNA encoding FLAG-tagged cyclin D1 to pLenti CMVTRE3G Puro DEST vector (Addgene ID: 27565). This vector was designated as TRE3G-D1. The transduction of MCF-7 cells was conducted by Lenti-virus infection as previously described (PMID: 22672904). Briefly, TRE3G-D1 plasmid DNA was introduced into HEK 293T cells by transient co-transfection with pMD2.G and psPAX2 with calcium phosphate precipitation. After 6 hrs post transfection, cell culture medium was replaced, and cells were allowed to grow for 36 hrs to produce viruses. The supernatant was then collected and filtered through a 0.45 ?m filter. After 48 hrs, MCF-7 cells stably expressing FLAG-cyclin D1 were selected by supplementing the medium with 2 μg/ml puromycin for 2 weeks. Immunofluorescence staining and Western blot were undertaken to determine the infection efficiency and expression levels of cyclin 1d, respectively [Correction added on 21 May 2015 after first online publication: the last six sentences were added in this paragraph.]. The target sequence for Dicer siRNA and negative control were purchased from Qiagen (Germantown, MD, USA). The luciferase reporter of wildtype (wt) DICER1 promoter and basic-pGL3 luciferase reporter with insertion of mutant (mut) DICER1 synthesized and purchased from GenePharma (Shanghai, China). siRNA transfections were performed as stated earlier by using Lipofectamine 2000 (Invitrogen, Waltham, MA, USA) 16. [Correction added on 21 May 2015 after first online publication: let-7 has been removed from the first sub-header of the Materials and Methods.]

### Cell viability and apoptosis ratio detection

Cells of different groups were adjusted to 3 × 10^3^ cells/well in the volume of 200 μl medium in 96-well plates; after 48 hrs, cells were cultured with 20 μl Methyl Thiazolyl Tetrazolium (MTT, M2128, SIGMA-ALDRICH, China) at 37°C for 4 hrs. Each group had five repeats on the same plate, and the results were presented as mean value ± SEM based on independent experiments. For apoptosis rates analysis, cells in the logarithmic phase were collected (5 × 10^4^ cells/ml) and cultured in 6-well dishes at 1 × 10^5^ cells/well. After treatment, the cells were suspended in 500 μl of 1× binding buffer, and then 5 μl Annexin V-FITC (BD Biosciences, San Diego, CA, USA) and 5 μl PI (20 μg/ml, BD Biosciences, San Diego, CA, USA) were added. The mixture was gently vortexed and incubated for 15 min. in the dark.

### Sphere-formation assays

Cells were plated in 6 cm ultra-low attachment dishes (Corning, Lowell, MA, USA); on the seventh day, the mammospheres were counted and measured under a low power field inverted microscope. The mammosphere formation efficiency was calculated as the percentage ratio of obtained spheres and plated cells 17, and was calculated by using the mean ± SD and *t*-test.

### Detection of miRNAs and protein expression

miRNAs expression levels were examined using real-time quantitative reverse transcriptase polymerase chain reaction as previously performed 11. Total protein was extracted, and the protein lysates were electrophoretically resolved on 10% SDS-PAGE and transferred to Nitrocellulose membrane. The membranes were incubated overnight with specific primary antibodies to cyclin D1 (1:1000; sc-20044; Santa Cruz Biotechnology, Dallas, Texas, USA), Dicer (1:800; sc-136981; Santa Cruz Biotechnology, Dallas, Texas, USA), p53 (1:2000; sc-6243; Santa Cruz Biotechnology, Dallas, Texas, USA) and Vinculin (1:2000; 4650; Cell Signaling, Danvers, MA, USA); the secondary antibodies were conjugated with HRP (1:2000; Santa Cruz Biotechnology, Dallas, Texas, USA) for 1 hr. The Western blot was scored as positive if the band of interest was present at the expected molecular weight.

### Immunofluorescence

Cells were planted in chamber 48 hrs prior to detection, and then fixed by incubating the slides in 10% formalin for 15 min. Cells were blocked in 2% goat serum (ab7481; Abcam, San Francisco, CA, USA); incubated with DICER1 (1:500; ab14601; Abcam, Burlingame, CA, USA) [Correction added on 21 May 2015 after first online publication: the value of DICER1 was added.] for at least 1 hr in PBST; incubated with Alexa Fluor® 488 (Goat antimouse IgG, Life Technologies, Bartlesville, OK, USA), for 30 min; washed in PBS, incubated for 10 min. with Hoechst (2 μg/ml, Life Technologies, Grand Island, NY, USA); and washed in PBS again. Fluorescence was visualized with a Leica (Leica Microsystems, Wetzlar, Germany) microscope (BD Biosciences).

### Luciferase assay

Cells of different groups were seeded at 50% confluency in a 24-well plate 16 hrs prior to transient transfection with the DICER1 luciferase reporter and basic-pGL3 luciferase reporter with mutant sites using FuGENE 6 Reagent (Roche, Indianapolis, IN, USA). Luciferase assays were performed at room temperature using an Autolumat LB 953 (Berthold Technologies, Oak Ridge, TN, USA) 48 hrs post-transfection and were normalized with β-gal results, as previously reported 18.

### Statistical analysis

All statistical analyses were performed using Excel. All data were represented as mean ± SD. Statistical analysis was conducted using the Student's *t*-tests. The significance of each value was determined, when *P* < 0.01.

## Results

### Reduced cyclin D1 is essential for let-7 induced cancer cell repression

**Figure 1:**
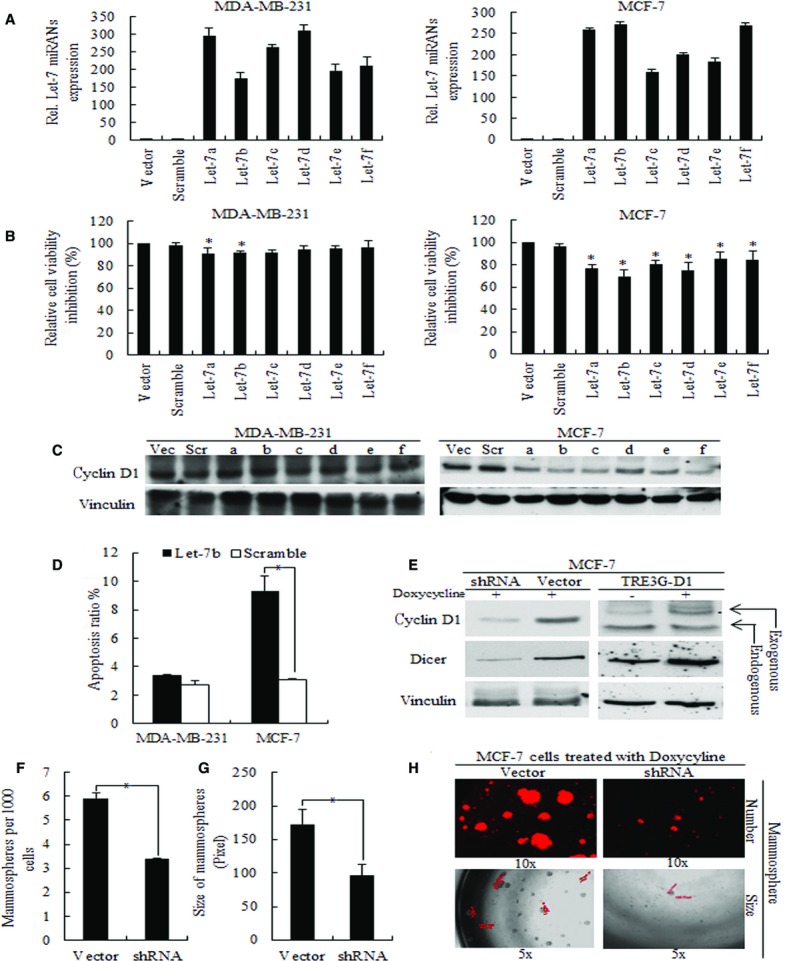
The proliferation inhibition and apoptosis ratio of let-7 overexpressed breast cancer cells were caused by decreased cyclin D1. (A) Let-7 expression levels in lentiviral-infected MCF-7 and MDA-MB-231 cells. (B) Let-7 inhibited cell proliferation of breast cancer cells, among which, let-7b exhibited the strongest effect, * p < 0.01. (C) Overexpression of let-7 miRNAs corresponded to decreased cyclin D1 expression in MCF-7 cells but not in MDA-MB-231 cells. (D) Let-7b also induced more cell apoptosis in MCF-7 cells, with little effects in MDA-MB-231 cells, * p < 0.01. (E) TET-treated TRE3G-cyclin D1 MCF-7 cells possesses exogenous extra cyclin D1, and RFP-based shRNA-cyclin D1 exhibited decreased cyclin D1 expression in MCF-7 cells; Dicer1 is positively correlated with cyclin D1 level. Down-regulation of cyclin D1 decreased both the number (F) and size (G) of mammospheres. (H) Representative images of mammospheres acquired from shRNA mediated cyclin D1 knockdown [Correction added on 21 May 2015 after first online publication: TET-induced was removed from the text.] in MCF-7 cells are shown.

In let-7-overexpressed MCF-7 and MDA-MB-231 cells (Fig.1A), we found that let-7 inhibited the cyclin D1 in MCF-7 cells only (Fig.1B). Furthermore, increased let-7 expression corresponded to reduced cell proliferation and increased apoptosis in MCF-7 cells only, indicating the essential roles of reduced cyclin D1 for let-7-regulated cell biology (Fig.1C and 1D). As let-7b exerted the most effective inhibition on cell growth, we focused on let-7b in the following studies. We also checked the roles of cyclin D1 in the self-renewal ability of breast cancer cells by using RFP-based [Correction added on 21 May 2015 after first online publication: TET-on was removed.] shRNA-cyclin D1 in MCF-7 cells (Fig.1E); results showed that knockdown of cyclin D1 inhibited both the stem cell numbers (Fig.1F) and mammosphere size (Fig.1G); representative images are shown in Figure1H.

### Let-7 expression level independent of DICER1 requires cyclin D1

**Figure 2:**
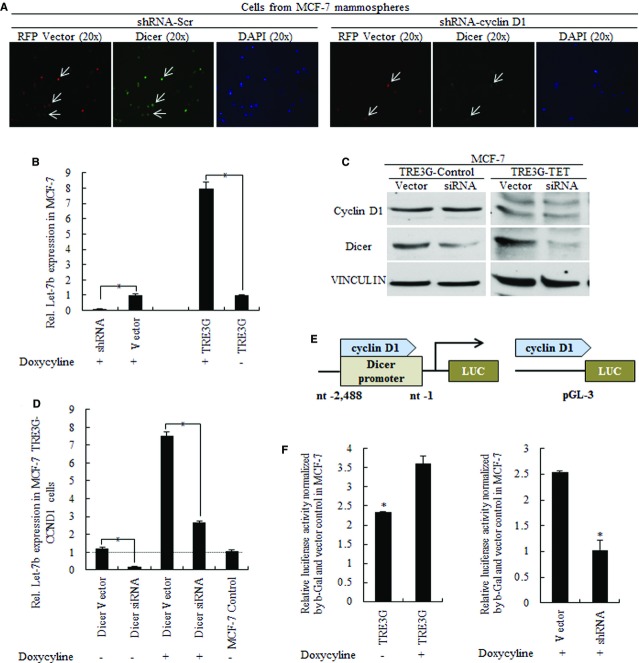
Cyclin D1 decreased let-7 expression through Dicer. (A) Immunofluorescent staining of Dicer (Green) in mammospheres from cyclin D1 knockdown MCF-7 cells, results showing that the decreased cyclin D1 achieved by shRNA-CCND1 (Red for RFP lentiviral) repressed Dicer expression level. (B) Let-7 expression was positively related with cyclin D1 expression, * p < 0.01. (C) Western blot results of Dicer siRNA-treated TRE3G-cyclin D1 cells. (D) Let-7 expression in cyclin D1-overexpressed MCF-7 cells was affected mainly by Dicer, * p < 0.01. (E) The illustration of cyclin D1 regulated Dicer-pGL3 Luc-vector. (F) Dicer promoter was regulated by cyclin D1 expression, * p < 0.01.

DICER1 is the critical regulator of miRNA processing, and cyclin D1 was demonstrated to regulate DICER1 and subsequent miRNAs expression 8. To confirm the relationship between cyclin D1 and let-7, we used TET-inducible cyclin D1-overexpressed MCF-7 cells and shRNA mediated cyclin D1 knockdown in MCF-7 cells, and found that DICER1 expression correlated with Tetracycline (TET)-induced cyclin D1 expression, as shown in Figure1E. Furthermore, using an immunofluorescent assay, we identified that deregulated DICER1 expression levels corresponded to cyclin D1 expression (Fig.2A). We then explored let-7b expression and found that increased cyclin D1 significantly increased let-7b expression level (Fig.2B), with exogenous cyclin D1 and DICER1 overexpressed (Fig.1E); inversely, in cyclin D1 knockdown MCF-7 cells, let-7b expression decreased (Fig.2B), with DICER1 expression being inhibited (Fig.1E).

### Deregulated Dicer responded to cyclin D1 regulation is critical for let-7 signature

We used DICER1 siRNA to identify the roles of DICER1 in let-7 processing of cyclin D1-overexpressed MCF-7 cells. Results showed that decreased DICER1 (Fig.2C) decreased let-7 expression and significantly abolished cyclin D1 induced let-7 expression (Fig.2D). Furthermore, we found that cyclin D1 effectively activated the DICER1 promoter activity, identifying the role of cyclin D1 in targeting and regulating DICER1 as downstream gene (Fig.2E and 2F).

### The regulatory pathway of p53/DICER1/let-7b in cell death response requires cyclin D1

**Figure 3:**
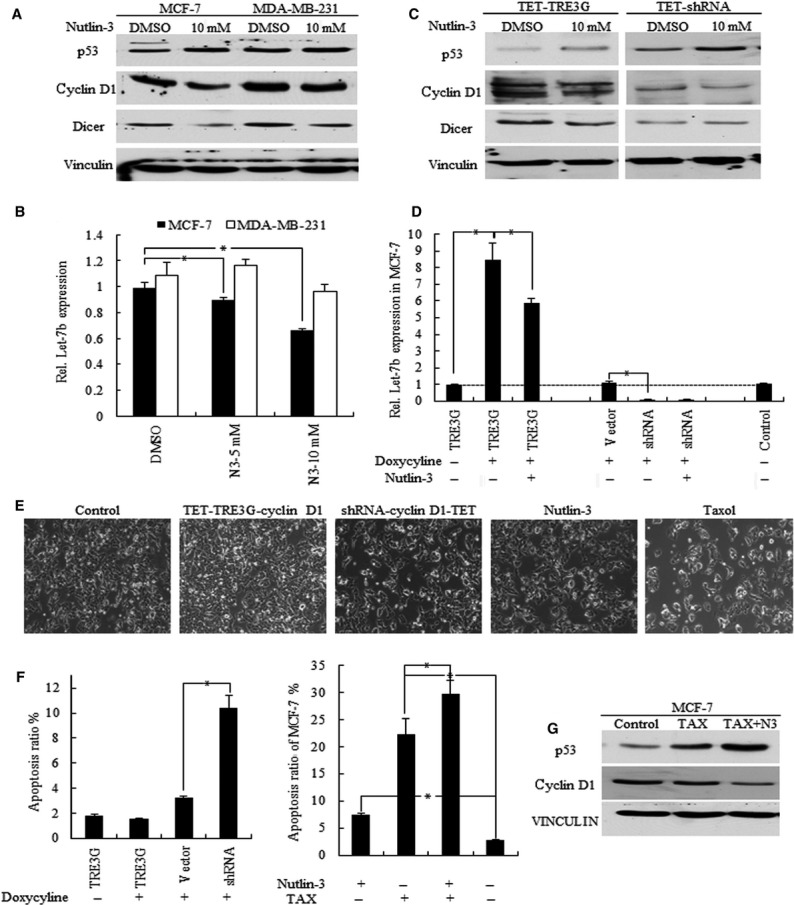
Let-7 signature variation in p53 and cyclin D1 regulated MCF-7 depend on Dicer. (A) Increased p53 by 10 μM of Nutlin-3 inhibited cyclin D1 and Dicer in MCF-7 but not MDA-MB-231 cells. (B) Let-7 was increased by overexpressed cyclin D1, and decreased in cyclin D1 knockdown cells, * p < 0.01. (C) p53 decreased endogenous cyclin D1 of TET treated TRE3G-cyclin D1-MCF-7 cells, and then decreased Dicer; however, in cyclin D1 knockdown cells, p53 failed to inhibited Dicer expression. (D) Nutlin-3 decreased let-7b expression in MCF-7 control and cyclin d1 overexpressed cells, but did not function in cyclin D1 knockdown cells, * p < 0.01. (E) Representative images of cell apoptosis of different groups are shown. (F) The knockdown of cyclin D1, increase in p53 and treatment with Taxol (TAX) all induced cell apoptosis, and Nutlin-3 sensitize cancer cells to TAX induced cell apopsosis, * p < 0.01. (G) TAX promoted cell apoptosis through regulating p53 and cyclin D1 levels, and N3 promoted this repression.

p53 interacted with cyclin D1 in controlling cancer cell biology, and we also found that increased p53 inhibited both cyclin D1 and DICER1 (Fig.3A), contributed to let-7 inhibition in MCF-7 cells (Fig.3B). However, in MDA-MB-231 cells, Nutlin-3 (N3), a molecule that induces p53 stabilization by inhibiting MDM2-dependent p53 degradation, did not affect let-7 expression effectively, with no significant changes of cyclin D1 level detected (Fig.3A and 3B). When treated with N3, p53 repressed the endogenous cyclin D1 expression, and reduced let-7 expression significantly in TET-treated TRE3G-MCF-7 cells but not in cyclin D1 knockdown cells (Fig.3C and 3D).

### Decreased let-7b indicates better results of N3 and Tax-induced cancer repression

**Figure 4:**
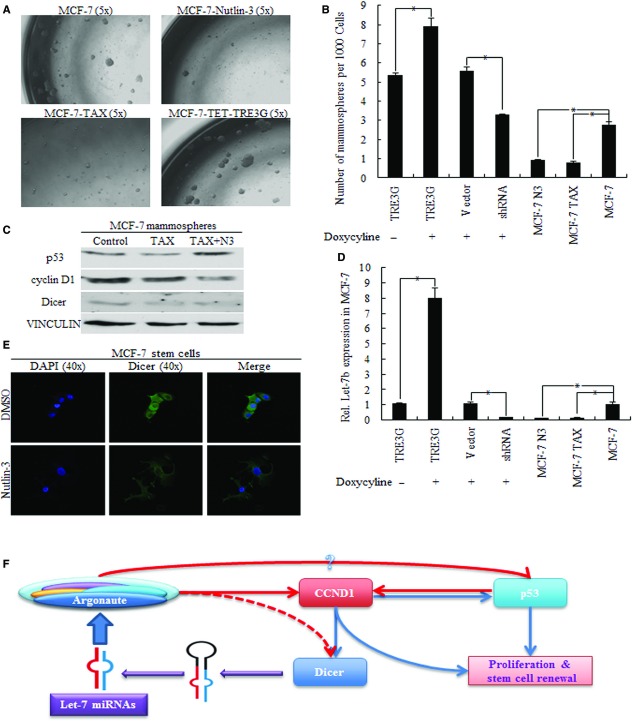
Nutlin-3 affected the let-7/cyclin D1 loop in breast cancer stem cells. (A) Representative images of first generation of mammospheres acquired from different groups. (B) Increased cyclin D1 increased mammosphere numbers, while both p53 and TAX treatment decreased sphere numbers, * p < 0.01. (C) Higher levels of cyclin D1 and Dicer were responsible for elevated let-7b; TAX and Nutlin-3 inhibited let-7b expression through down-regulating cyclin D1 and Dicer. (D) Relative let-7b expression level in MCF-7 of different groups, * p < 0.01. (E) Both nuclear and cytoplasmic Dicer were inhibited by increased p53 induced by Nutlin-3. (F) Let-7 inhibited DICER1 expression partially through cyclin D1 inhibition, forming let-7/cyclin D1/DICER1 negative feedback loop.

Decreased let-7b was related to p53-induced cell apoptosis, indicating better anticancer effects of chemotherapy (Fig.3E and 3F). Exactly, 10 μM of N3 and 10 nM of TAX induced cells apoptosis significantly through regulating p53 and cyclin D1 (Fig.3F and 3G). N3 and TAX also decreased the self-renewal of cancer stem cells, while cyclin D1 increased mammosphere numbers (Fig.4A and B). Increased p53, together with deceased cyclin D1 and DICER1 in mammospheres were responsible for let-7 inhibition (Fig.4C and D). We also confirmed the positive correlation between cyclin D1 and DICER1 in cancer cells acquired from N3-treated MCF-7 mammospheres (Fig.4E). cyclin D1 is the key factor for the let-7/Dicer regulatory loop, regulating cell death and self-renewal ability of stem cells, as was shown in Figure4F. Tumour suppressive let-7 acted as the indicator in p53 regulated chemotherapy response, which was achieved through the let-7/cyclin D1/DICER1 loop.

## Conclusions

Let-7 miRNAs were traditionally regarded as regulators of downstream mRNAs; however, recent studies discovered some regulatory loops between let-7 and its targeted genes 19. Cyclin D1 was one of the traditional oncogenes in the regulation of cancer malignancy, and more recently, was regarded to miRNA genesis and maturation though regulating DICER1, the key enzyme for miRNAs processing 8,20. Let-7 targets and degrades cyclin D1 through the post-transcriptional regulation of mRNA, and therefore, we wondered if cyclin D1 could regulate upstream let-7 through DICER1, forming a feedback loop.

In this research, we found that cyclin D1 could increase the let-7 expression levels, which was assisted by DICER1. SiRNA-mediated knockdown of DICER1 in MCF-7 cells led to defects in let-7 production, proving the crucial roles of DICER1 in cyclin D1-induced let-7 expression alteration. Tumour inhibition caused by p53 was referred to DICER1 repression. In conclusion, cyclin D1 induces DICER1 and thereby promotes the maturation of let-7 miRNA, and then drive cancer progression in part *via* miRNA biogenesis. We then explored the roles of p53 in cyclin D1-regulated miRNA expressions, results showing that p53-repressed cyclin D1 expressions contributed to let-7 depression. The interactions between cyclin D1 and p53 also functioned in N3 and TAX-induced cell apoptosis and suppression of self-renewal of stem cells, affecting let-7 expressions. In conclusion, we confirmed the feedback loop between let-7 and its downstream cyclin D1, which was achieved by DICER1. Also, the existence of cyclin D1 was crucial for chemotherapy-induced let-7 alteration.

Dicer converts inactive hairpin-structured pre-miRNA into the active single stranded form, then influence multiple cellular functions 8,10,21. Oncogenic cyclin D1 could promote Dicer, and then induced let-7 maturation, forming the let-7/DICER1 and let-7/cyclin D1 regulatory loop. We identified in this study that cyclin D1 regulated let-7 could act as useful indicator in evaluating chemotherapy response. Our unpublished data also indicated that ESR1 could be involved in let-7/cyclin D1 feedback loop through interactions with DICER1 or LIN28 respectively. Although acting as tumour suppressor, the repressed let-7 in cells receiving anticancer treatments may indicate better effects, which further indicates the complex mechanisms of cellular miRNAs functions.
